# Identifying the Neural Substrates of Procrastination: a Resting-State fMRI Study

**DOI:** 10.1038/srep33203

**Published:** 2016-09-12

**Authors:** Wenwen Zhang, Xiangpeng Wang, Tingyong Feng

**Affiliations:** 1 Key Laboratory of Cognition and Personality, School of Psychology, Southwest University, Chongqing 400715, China; 2 Key Laboratory of Cognition and Personality, Ministry of Education, Southwest University, Chongqing 400715, China

## Abstract

Procrastination is a prevalent problematic behavior that brings serious consequences to individuals who suffer from it. Although this phenomenon has received increasing attention from researchers, the underpinning neural substrates of it is poorly studied. To examine the neural bases subserving procrastination, the present study employed resting-state fMRI. The main results were as follows: (1) the behavioral procrastination was positively correlated with the regional activity of the ventromedial prefrontal cortex (vmPFC) and the parahippocampal cortex (PHC), while negatively correlated with that of the anterior prefrontal cortex (aPFC). (2) The aPFC-seed connectivity with the anterior medial prefrontal cortex and the posterior cingulate cortex was positively associated with procrastination. (3) The connectivity between vmPFC and several other regions, such as the dorsomedial prefrontal cortex, the bilateral inferior prefrontal cortex showed a negative association with procrastination. These results suggested that procrastination could be attributed to, on the one hand, hyper-activity of the default mode network (DMN) that overrides the prefrontal control signal; while on the other hand, the failure of top-down control exerted by the aPFC on the DMN. Therefore, the present study unravels the biomarkers of procrastination and provides treatment targets for procrastination prevention.

It is important for people to initiate and adapt information processing through temporal context. Deficits in this ability would make individuals put off scheduled things until deadline, and would further affect one’s daily life, working business^1^ or even public policy^2^. In psychology, this phenomenon is often defined as “procrastination” that individuals “voluntarily delay an intended course of action despite expecting to be worse off for the delay”^3^. Procrastination has become a prevalent problematic behavior that brings serious consequences to individuals who suffer from it. It has been reported that approximately 20% of the population are affected by chronical procrastination^4^. Existing evidence have documented that procrastination is associated with poor mental health^5^ and low life satisfaction^6^.

The extensive literature concerning procrastination has attributed this phenomenon to cognitive and affective factors^7^. Several models are proposed to interpret the psychological mechanisms subserving procrastination, which predominantly characterize procrastination as a failure of temporal-based self-control^8^,^9^ that may result from problematic inhibition of the motive force of emotion^10^. Sirois proposed a cognitive escape hypothesis, according to which procrastinators manifest avoidant cognitive tendencies to promote immediate emotion regulation at the cost of long-term goals^11^. However, despite the increasing attention from researchers on procrastination, the neural substrates underpinning this phenomenon have been understudied.

In general, self-control is underpinned by two interacting brain systems: a cognitive control system and an affective processing system^12^. The cognitive control system, located mainly in the prefrontal cortex^13^, functions to exert top-down signals to facilitate goal-directed behaviors^14^. The affective processing system is responsible for responding to emotional stimuli and self-related information^15^,^16^, and this system is supported by the limbic system^17^ and the default mode network (DMN)^18^. Interestingly, empirical evidence has demonstrated that, procrastination is associated with the failure of cognitive control^11^,^19^. On the other hand, one study suggests that procrastination is correlated with bad emotional states^20^, and individuals with procrastination tend to focus on short-term mood repair, showing a temporal disjunction between the present and future selves^21^,^22^. Based on these findings, we hypothesized that procrastination is attributed to the operations of the prefrontal cortex, the limbic system and/or the DMN. That is, the regional activity in prefrontal cortex would negatively correlate to behavioral procrastination, while the activity in limbic system and/or the DMN would be positively associated with procrastination. Furthermore, procrastination might be determined by the interactions between cognitive-related regions and affective-related areas.

In the present study, we employed resting-state fMRI (RS-fMRI) to unravel the neural substrates of trait procrastination. RS-fMRI is a relatively new method which is useful for the investigation of the intrinsic functional architecture underlying trait-like personality and behaviors^23^. To begin with, we performed the regional homogeneity (ReHo) analysis to detect regional activity correlating to procrastination. Then amplitude of low-frequency fluctuation (ALFF) was calculated to retest the results of the ReHo analysis. Both ReHo and ALFF are effective methods to detect spontaneous neural activity^24^,^25^. To further test the hypothesis that procrastination could be attributed to brain interactions, we estimated the functional connectivity (FC) patterns and correlated them with the behavioral procrastination.

## Results

### ReHo and ALFF results

**Figure 1:**
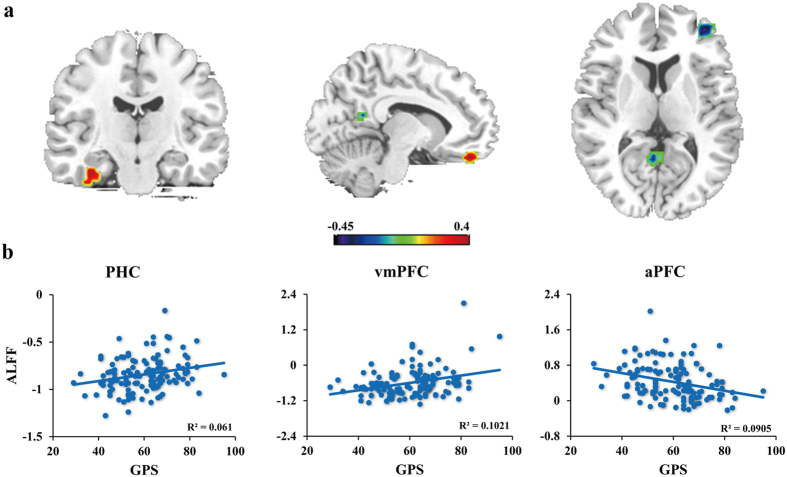
Spontaneous regional activity subserving procrastination. Panel a displays the correlation between procrastination and regional homogeneity (ReHo) values. Color bar represents a scale of *r* value. The significance level was set at *p* < 0.05 (corrected). Panel b shows the correlations between procrastination and regional ALFF values. GPS refers to the general procrastination scale. PHC = parahippocampal cortex, vmPFC = ventromedial prefrontal cortex, aPFC = anterior prefrontal cortex.

The results of the ReHo analysis is depicted in Fig. 1a. The regions whose activity showing positive correlations with the GPS scores were the parahippocampal cortex (PHC) (x = −36, y = −18, z = −30) and the ventromedial prefrontal cortex (vmPFC) (x = 9, y = 42, z = −24). While those negatively correlated with procrastination were the anterior prefrontal cortex (aPFC) (x = 39, y = 48, z = 6) and the posterior cingulate cortex (PCC) (x = 6, y = −57, z = 15). The results were AlphaSim corrected at the level of *p* < 0.05.

The zALFF values were extracted from the four regions mentioned above. The partial correlation analyses revealed that the zALFF values in the aPFC negatively correlated with the GPS scores, *r* = −0.330, *p* < 0.001, *df* = 128, two-tailed. Meanwhile, the zALFF values in the PHC and the vmPFC showed positive correlations with procrastination (*r* = 0.293, *p* < 0.001; *r* = 0.334, *p* < 0.001; *df* = 128, two-tailed). However, the correlation between the PCC zALFF values and procrastination was not significant, *r* = −0.162, *p* > 0.05, *df* = 128, two-tailed (Fig. 1b). In total, the three variables could account for 20.6% variance of procrastination.

### Functional connectivity analysis

**Figure 2:**
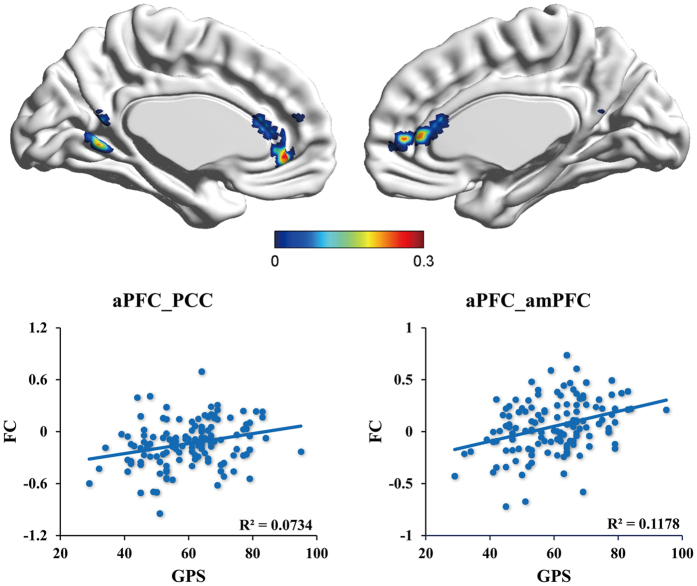
The aPFC-seed functional network underpinning procrastination. Results in the upper panel was corrected using AlphaSim correction at the level of *p* < 0.05. Color bar represents a scale of *r* value. PCC = posterior cingulate cortex, amPFC = anterior medial prefrontal cortex, aPFC = anterior prefrontal cortex, FC = functional connectivity. aPFC_PCC refers to the functional connectivity between aPFC and PCC.

The connectivity-behavioral correlation analysis revealed the neural networks underlying procrastination. For the aPFC-seed analysis, the functional connectivity between the aPFC and the anterior medial prefrontal cortex (amPFC) (x = −3, y = 30, z = 12), and between the aPFC and the PCC (x = −6, y = −57, z = 3) was positively correlated with procrastination (Fig. 2).

**Figure 3:**
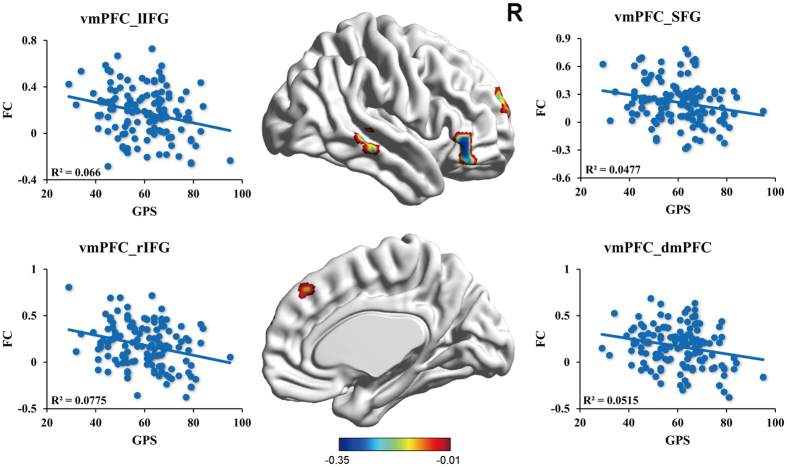
The vmPFC-seed functional network underpinning procrastination. Color bar represents a scale of *r* value. The significance level was set at *p* < 0.05 (corrected). SFG = superior frontal cortex, rIFG = right inferior frontal gyrus, lIFG = left frontal cortex, vmPFC = ventromedial prefrontal cortex, dmPFC = dorsomedial prefrontal cortex, FC = functional connectivity. vmPFC_SFG refers to the functional connectivity between vmPFC and the SFG.

For the vmPFC-seed connectivity, the GPS scores showed significant negative correlations with the connectivity between the vmPFC and several regions, including the dorsomedial prefrontal cortex (dmPFC) (x = −3, y = 36, z = 39), the superior prefrontal cortex (x = 12, y = 57, z = 24), the bilateral inferior prefrontal cortex (IFG) (x = −54, y = 33, z = 6; x = 51, y = 33, z = −3) and the right middle temporal cortex (x = 60, y = −33, z = 0) (Fig. 3). However, for the PHC-seed connectivity, the GPS scores showed no significant correlation with the connectivity between the PHC and any other regions. All these results were AlphaSim corrected at the significance level of *p* < 0.05.

## Discussion

Employing RS-fMRI, the present study aimed to explore the neural substrates subserving procrastination. Results from ReHo combined ALFF analyses showed that the behavioural procrastination was positively correlated with the regional activity of the PHC and the vmPFC, while negatively correlated with the activity of the aPFC. In addition, increased functional connectivity between the aPFC and the PCC, the amPFC was associated with increased procrastination tendency. Meanwhile, the functional connectivity between the vmPFC and several other regions including the prefrontal cortex, the medial temporal cortex was negatively associated with procrastination.

Previous studies have demonstrated the engagement of the PHC in the processing of episodic memory^26^ and emotional stimuli^27^. Aminoff *et al*. proposed an integrative account, whereby the PHC is sensitive to contextual association and reflects activation of the relevant stored contextual representation^28^. According to this account, the PHC could function to incorporate the current context with long-term associations of the context built up in memory. This is consistent with the findings that the PHC is a key region responsible for episodic or semantic prospection^29^,^30^. Intriguingly, cumulative evidence have showed that episodic future thinking is associated with procrastination^31^, and imaging negative future events made individuals incline to choose immediate rewards^32^. Combined, the positive correlation between the regional activity of the PHC and procrastination suggested that procrastination is accompanied by increased negative episodic prospection encoded in the PHC.

Meanwhile, the extensive literature has assigned to the vmPFC responsibility for decision making^33^,^34^. Rushworth *et al*. argued that the vmPFC is a key region involved in value-guided decision making to pursue the proper option^35^. In addition, the vmPFC is also considered as a hub region that integrates multiple-source information, e. g., episodic memory, value evaluation, self-directed cognition and emotion^36^. In the model proposed by Bechara^37^, the regional activity of the vmPFC is modulated by top-down control signal exerted by the prefrontal cortex, and on the other hand influenced by a bottom-up affective process arises from the impulsive system (e. g., amygdala and the hippocampal formation). According to this model, decision making in vmPFC is determined by the trade-off between cognitive control and affective processing.

Intriguingly, the PHC and the vmPFC are core regions of the default mode network and could interact with each other to facilitate decision making based on mnemonic scene construction^18^. In procrastination, greater activity of the PHC evoked by negative episodic prospection might bias the decision-making processing in the vmPFC towards immediate satisfaction. This process would further affect the activity of several other regions. In particular, greater negative vmPFC-seed connectivity with the dmPFC, the bilateral IFG and some others was associated with increase in procrastination tendency. The dmPFC and the IFG have been demonstrated as core regions of cognitive control. To specify, the dmPFC is engaged in mentalizing^38^ and evaluation of self-referential stimuli^39^. Recent studies have proposed that the dmPFC plays a critical role in intentional control, supporting top-down emotion regulation^40^ and voluntarily refraining from planned behaviors^41^. These findings suggest that the dmPFC is a key region that functions to exert internally-generated self-control. In addition, some pooled studies have documented the involvement of the bilateral IFG in inhibitory and attention control^42^,^43^. Tabibnia *et al*. reported that the IFG play a cardinal role in both the “cold” cognitive inhibitory control and the “hot” affective self-control^44^. The present findings that increased negative correlation between the vmPFC and these regions gave rise to procrastination indicated that hyper-activity of the vmPFC could hamper the activity of the prefrontal cortex, and override the top-down control signals to focus on short-term satisfaction (e.g., mood repair). This is consistent with the argument that in behaviors such as drug addiction, strong bottom-up signals triggered by the impulsive system could hijack the goal-driven cognitive signal and result in a failure of self-control^37^.

The present study also found that the activity of the aPFC was negatively correlated with procrastination, that is, increased activity of the aPFC inhibited procrastination. The activity of the aPFC has been observed in many high-level cognitive tasks, such as long-term planning and reasoning^45^,^46^. Meanwhile, the aPFC also plays an important role in prospective memory^47^, a form of memory targeting planed actions and future intentions. To specify, it supports the recollection of contextual information^48^ and encoding of future intentions^49^. Employing transcranial magnetic stimulation (TMS), Volman *et al*. found that the inhibition of the aPFC could impair control over social emotional actions; therefore highlighted the involvement of this region in emotional control^50^. A probable integrative account is that the aPFC functions to coordinate cognitive and affective information in order to initiate temporal-based cognitive control. Therefore, with the recruitment of the aPFC, the low-score individuals could focus on future goals to reduce procrastination; while individuals scored higher in procrastination would show deficits in this region and prefer short-term intentions. This is consistent with the observation that procrastination is predicted by a reduced focus on the future^9^ and that long-term preference is associated with greater aPFC activity^51^.

Functional connectivity analysis revealed that the connectivity between the aPFC and the PCC, the amPFC was positively correlated with procrastination. The results suggested that the inhibitory effect of the aPFC on procrastination was achieved by suppressing the activity of the PCC and the amPFC. The literature have demonstrated that the PCC and the amPFC are hub regions of the DMN and are responsible for the processing of personal significance, mentalizing, and evoked emotion^18^. In general, deactivation of the DMN activity facilitates goal-directed behaviors by inhibiting the interference from distractors^52^. Otherwise, compromised DMN suppression impairs cognitive functions, and hyper-activity of the DMN was observed in several mental disorders^53^. In current study, greater negative correlation between aPFC and the DMN was associated with lower procrastination tendency, suggesting that the aPFC could exert cognitive control over the DMN to help individuals focus on task goal in the future. However, absence of aPFC inhibition on DMN, revealing in positive correlation between them, would result in procrastination.

Combined those findings, the present study suggested that cognitive control failures in procrastination, reflecting in the overwhelming priority of the DMN activity over inhibitory control exerted by the prefrontal cortex, and/or reflecting in the absence of the aPFC control on the DMN. This is consistent with the balance model of self-control^54^, which suggest that self-control failures once impulses overwhelm prefrontal control or PFC function is impaired. Note that the ReHo results also showed a negative correlation between the regional activity of the PCC and the procrastination. Considering the engagement of the PCC in reward and value evaluation^55^,^56^, it might be that greater procrastination is associated with lower personal value. Whilst, the PCC is of heterogeneity, different parts of the PCC function differently according to task demands^57^,^58^. Therefore, further evidences are needed to interpret the role of the PCC in procrastination. However, since the correlation between the activity of the PCC and procrastination was not confirmed in ALFF, we therefore did not considered it as a core neural substrate of procrastination.

By employing resting-state fMRI, the present study demonstrated that the key process underpinning procrastination is the trade-off between cognitive control and affective processing. Procrastination occurs while the aPFC lose control over the DMN, and hyper-activity of the PHC and the vmPFC biases one’s attention towards immediate/short-term satisfaction and overrides the activity of brain regions responsible for inhibiting internal/external distracting stimuli. In this case, our findings revealed the neural substrates subserving procrastination and provided new perspectives in understanding the mechanisms behind this phenomenon. We propose that cognitive training programs targeting on the plasticity of prefrontal cortex might help to prevent procrastination. In addition, the study suggests that the aPFC could serve as a target for physical interventions, such as the TMS and the transcranial direct current stimulation (tDCS)^59^ in the treatment of procrastination.

## Methods

### Participants

The study was approved by the Institutional Review Board of the Faculty of Psychology, Southwest University. We had obtained appropriate ethics committee approval for the experiment reported, and followed the guidance of the APA requirements of human subjects. One hundred and thirty-two healthy students (95 females and 37 males) from Southwest University, China, participated in the experiment with compensation of 60 yuan (RMB). The mean age of the participants was 21.41 ± 2.06. All participants were native Chinese speakers with no history of mental diseases. Signed informed consent was obtained from each participant prior to the experiment.

### The General Procrastination Scale (GPS)

The present study adopted the General Procrastination Scale (GPS) designed by Lay^60^ to measure trait procrastination. The GPS is a widely used scale which has been demonstrated as a reliable and valid measurement of procrastination in daily tasks. It contains 20 items (e.g., “I often find myself performing tasks that I had intended to do days before”, “I do not do assignments until just before they are to be handed in”, etc.). Participants rated themselves on a scale of 1–5 from “extremely uncharacteristic” to “extremely characteristic”. The reliability of the GPS was assessed using the coefficient Cronbach’s alpha, with a score of 0.883. The mean score of the GPS in current study was 59.27 ± 1.69.

### fMRI Data Acquisition

Imaging data was collected with a Siemens Trio 3.0 T scanner in the Key Laboratory of Cognition and Personality at Southwest University, China. T2-weighted functional images were acquired in an interlaced way along the AC–PC line with a T2-weighted EPI sequence of 33 axial slices (TR = 2000 ms, TE = 30 ms, flip angle = 90, matrix size = 64 × 64, slice thickness = 3 mm) with 0.6 mm inter-slice gap. Each scan session consisted of 240 images. At the end of the experiment, anatomical images were also collected using a GR\IR sequence (TR = 2530 ms, TE = 3.39 ms, flip angle = 7, acquisition matrix = 256 × 256).

### fMRI Data Preprocessing

Preprocessing was performed using Data Processing Assistant for resting-state fMRI (DPARSF). The first 10 images were discarded to avoid T1 saturation effects and to allow the BOLD signal to stabilize. Then the data were corrected for differences in slice-timing and spatially realigned to the first volume of the data set. Individual structural images were co-registered to the mean functional image and then segmented into grey matter, white matter, and cerebrospinal fluid using “new-segment and DARTEL”. After this, nuisance covariates including Friston-24 head motion parameters, white matter signal and cerebrospinal fluid signal were regressed out. Then liner trends were removed and filtering was performed using 0.01–0.08 Hz band-pass to reduce the effects of low-frequency drifts and high-frequency noise. Finally, the images were normalized to the MNI space (voxel size: 3 mm3) with the DARTEL tool and smoothed with a Gaussian kernel of 6 mm full-width at half-maximum.

For each participant, the following analyses were performed within a gray matter mask. Only these voxels with a probability higher than 0.4 in the SPM8 gray matter mask were included. In total, there were 49483 voxels within this mask. The significance level of the fMRI data was first set to *p* < 0.005 at the individual voxel level. Then we performed AlphaSim program for multiple comparison. The uncorrected *p* threshold was set to 0.005, and we ran 1000 Monte Carlo simulations with Gaussian filter width in 6 mm and Cluster connection radius in 5 mm. The cluster size for multiple comparison at the corrected level of *p* < 0.05 was 21 contiguous voxels. We then used this corrected AlphaSim threshold as the significance level for the following analyses.

### Regional Homogeneity Calculation

To detect regional activity that correlated to procrastination, we performed the ReHo analysis using the REST software (http://restfmri.net/). The procedures were similar to those used in previous studies^24^. This analysis was performed using the images without smoothing in pre-processing. A Kendall’s coefficient of concordance (KCC) was assigned to a given voxel, and we calculated the KCC of its time course with those of the 26 surrounding neighbours. For each subject, the same procedure was implemented to obtain the individual KCC map. Then the individual KCC map was divided by its own mean ReHo value and transformed using Fisher’s r-to-z transformation to produce z map.

A group-level whole-brain multiple regression analysis was performed to correlate the zReHo maps with the GPS scores. Notably, the demographic variables such as age and gender were regressed as covariates to exclude any confounds.

### Amplitude of Low Frequency Fluctuation Calculation

ALFF was performed using REST software (http://restfmri.net/). Using fast Fourier transform, the time series of the voxels were transformed to frequency domain to obtain the power spectrum. Then the power spectrum was square rooted and averaged within the low-frequency range (0.01–0.08 Hz) to get the ALFF value. For the purpose of standardization, the ALFF of each voxel was divided by the global mean ALFF value for each subject. The resulting ALFF maps were then transformed into z values using Fisher’s r-to-z transformation to produce *z* maps.

To test the reliability of the ReHo results, we extracted the zALFF values of the resulting regions in ReHo analysis and a partial correlation was performed to correlate them with the GPS scores. The seed regions were defined around the local peak with a 6 mm radius sphere respectively. Age and gender were controlled as covariates in the analysis.

### Functional Connectivity Calculation

To detect the neural networks subserving procrastination, we performed a functional connectivity (FC) analysis. Voxel-wise FC was performed to calculate correlation coefficients between seed region and all other voxels in the brain. According to the ReHo and ALFF results, three seed regions were defined as regions of interest: the aPFC, the parahippocampal cortex (PHC) and the vmPFC. Connectivity maps were produced by extracting the mean time series from the three seed regions separately and then calculating its correlation coefficients with the rest voxels of the brain. Subsequently, for each seed region, the resulting FC map was transformed into z values using Fisher’s r-to-z transformation.

To identify the specific functional connectivity underpins procrastination, we performed an additional analysis to correlate the behavioral procrastination with the voxel-wise FC maps. This analysis was performed after controlling age and gender.

## Additional Information

**How to cite this article**: Zhang, W. *et al*. Identifying the Neural Substrates of Procrastination: a Resting-State fMRI Study. *Sci. Rep.* **6**, 33203; doi: 10.1038/srep33203 (2016).
